# Synthesis, structural characterization and Cu(ii) adsorption behavior of manganite (γ-MnOOH) nanorods

**DOI:** 10.1039/c9ra09652c

**Published:** 2019-12-24

**Authors:** L. F. Cano-Salazar, A. Martínez-Luévanos, J. A. Claudio-Rizo, F. R. Carrillo-Pedroza, S. M. Montemayor, J. R. Rangel-Mendez

**Affiliations:** Facultad de Ciencias Químicas, Universidad Autónoma de Coahuila V. Carranza s/n 25280 Saltillo Coahuila Mexico aml15902@uadec.edu.mx +52-84-4169213 +52-84-41383973; Facultad de Metalurgia, Universidad Autónoma de Coahuila Carretera 57 Km. 4.5 Zona Universitaria C.P. 25750 Monclova Coahuila Mexico; Centro de Investigación en Química Aplicada Blvd. Enrique Reyna Hermosillo No. 140 C.P. 25294 Saltillo Coahuila Mexico; Environmental Sciences Division, Instituto Potosino de Investigación Científica y Tecnológica A. C. San Luis Potosí, S. L. P. Mexico

## Abstract

New alternatives for the removal of transition metal ions that present an environmental risk are required. The chemical adsorption of these ions on surfaces with chemisorbent properties represents a promising area of research. In this work, manganite (γ-MnOOH) nanorods were synthesized, with a surface area of 20.22 m^2^ g^−1^, pore size of 32.18 nm and pore volume of 0.1627 cm^3^ g^−1^. After chemical and structural characterization of the manganite sample, it was evaluated as an adsorbent of Cu(ii) from aqueous solution. The equilibrium adsorption data were well fitted by the Langmuir isotherm, and the results indicated that the maximum adsorption capacity of Cu(ii) was 11.926 mg g^−1^. Cu(ii) ion adsorption on the manganite surface is a spontaneous and exothermic process (Δ*G*°< 0 and Δ*H*°< 0). The negative value of Δ*S*° suggests the stability of the adsorption process without structural change at the manganite–aqueous solution interface. A scheme for chemisorption of Cu(ii) ions on the hydroxylated surface of manganite is proposed.

## Abstract 2

Manganite (γ-MnOOH) nanorods were synthesized and Cu(ii) adsorption on their hydroxylated surface was a spontaneous process (Δ*G*° < 0).

## Graphical abstract

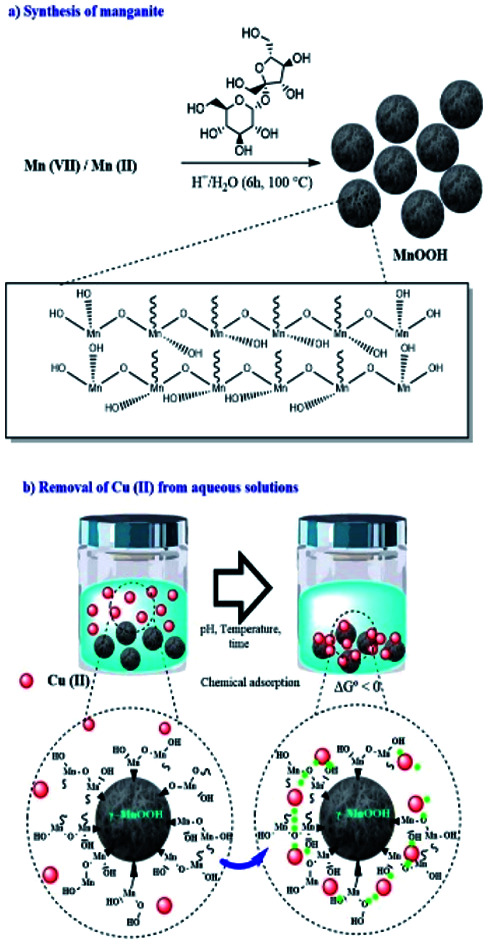

## 1. Introduction

The contamination of water by toxic heavy metals through the discharge of industrial wastewater is a worldwide environmental problem.^1–3^ Heavy metals are not only biodegradable but also tend to accumulate in living organisms causing several health effects.^4,5^ Copper ions are the most common heavy metals present in water, however, Cu(ii) is essential for all living organisms as a dietary source and a nutritional element, found in the vital parts of several enzymes, mainly.^6^ The acute exposure to doses of Cu(ii) higher than 100 ppm leads to severe mucosal irritation, widespread capillary damage and renal damage, central nervous problems followed by depression, gastrointestinal irritation and possible necrotic changes in the liver and kidney.^7^

With this in mind, any process or container involving copper could contaminate products such as food, water or drinks. The potential sources of Cu(ii) in industrial effluents include metal cleaning and plating, fertilizer industry, paints and pigments, municipal and storm water run-off.^8^ Thus, the removal of Cu(ii) from contaminated water has received increasing attention. A high number of approaches have been adapted and practiced to ensure the environmental safety against copper, the most widespread methods being used are: chemical precipitation, chelation/complexation, ion exchange, membrane filtration, ultrafiltration, reverse osmosis, electrodialysis, flotation, among others; all these methods have disadvantages as expensive chemical requirements, energetic costs, sludge disposal issues, among others.^9–11^ Due to this, the processes involving chemical adsorption have become very important alternatives, owing to their cheap cost effectiveness and the high-quality of the produced treated effluents.^12,13^ Among available adsorbents, metal oxides, including ferric oxides, manganese oxides, aluminum oxides, magnesium oxides and cerium oxides, are classified as promising surfaces for heavy metal removal from aqueous systems. Recent studies suggested that several oxides as goethite, maghemite, magnetite and hydrous manganese dioxides exhibit very favorable adsorption to heavy metals in terms of high capacity and selectivity, which may result in deep removal of toxic metals required for strict regulations.^14^

Manganite (γ-MnOOH) could be classified as a potentially interesting adsorbent for metallic cations, due to its crystal structure and chemical composition. For this reason, the aim of this work is to synthesize γ-MnOOH (manganite) with physical and chemical characteristics to be studied as an adsorbent of Cu(ii). Based on our knowledge, this is the first time that manganite is evaluated as an adsorbent of Cu(ii) ions.

## 2. Experimental

### 2.1. Materials

Chemical: potassium permanganate (KMnO_4_), sucrose (C_12_H_22_O_11_), manganese sulfate (MnSO_4_·H_2_O) and nitric acid (HNO_3_) were used to synthesize the manganite (γ-MnOOH). Hydrochloric acid (HCl) and sodium hydroxide (NaOH) were used for pH control. Copper sulfate (CuSO_4_·5H_2_O) was used as the target pollutant. Potassium permanganate and sucrose were obtained from Faga Lab. Manganese sulfate and sodium hydroxide were purchased from Jalmek, copper sulfate from Sigma Chemical Co. (St. Louis, MO., USA) and hydrochloric and nitric acids from Fermont. All chemical reagents were of analytical grade, used as received, without further purification.

### 2.2. Synthesis and characterization of manganite

Manganite (γ-MnOOH) was prepared at 100 °C according to the following procedure reported by Crisostomo *et al.*:^15^ a solution of KMnO_4_ (5.89 g) in 200 mL of deionized water was slowly added to a stirred solution of sucrose (2 g), MnSO_4_·H_2_O (4.40 g), 30 mL of water and 3 mL of HNO_3_. The resultant solution was refluxed under constant stirring for 6 hours. After hot filtration, the brown solid product was heavily washed with deionized water and air-dried.

The crystalline structure of the sample was analyzed by X-ray diffraction (XRD) using a Siemens D5000 X-ray diffractometer equipped with a Cu Kα X-ray source (*λ* = 1.54 Å). The sample was also characterized by Fourier transform infrared spectroscopy using a PerkinElmer Spectrum GX equipped with an Attenuated Total Reflectance accessory (ATR). A total of 10 scans per sample were carried out. For the determination of specific surface area, pore volume, and pore diameter, the sample was outgassed in a Beckman Coulter SA-PREP at 80 °C for 2 hours; after that, the sample was degassed in a Beckman Coulter SA-3100 surface area analyzer measuring the Brunauer–Emmett–Teller (BET) surface area of manganite. BET surface area was obtained from nitrogen adsorption data at −196 °C.

The thermal behavior of the manganite was evaluated by gravimetric thermal analysis (TGA), using a PerkinElmer TGA-4000 thermoanalyzer. The operating conditions used were a temperature range from 30 to 800 °C, a heating rate of 20 °C min^−1^, using nitrogen as inert atmosphere. Finally, the morphological analysis of the manganite was carried out using a FE-SEM 7401-F field emission scanning electron microscopy. The operating conditions were voltage acceleration of 2.0 kV and working distance of 3 mm. Chemical binding was analyzed by X-ray photoelectron spectroscopy (XPS) performed with a Thermo Scientific equipment under high vacuum (9.5 × 10^−9^ mbar) operating with Al Kα radiation at 12 kV and 40 W, each sample was sputtered 15 seconds for a better analysis of the surface.

### 2.3. Cu(ii) adsorption experiments

Adsorption kinetics of Cu(ii) on the manganite surface was carried out by studying the effect of the contact time and the pH of the solution. The effect of contact time on the adsorption of Cu(ii) from aqueous solutions was investigated using an initial concentration of 25 mg L^−1^ of copper. In addition, 200 mg of manganite were mixed with 50 mL of aqueous solutions containing Cu(ii), then, the mixtures were shaken (200 rpm). The contact time for the kinetics experiments was from 0 to 120 minutes. Suspensions were filtrated and copper concentration was measured with a Solar S4 Thermo Electron Corporation atomic absorption spectrophotometer. The effect of pH on the adsorption phenomenon was studied by adding either 0.1 M HCl or NaOH in Cu(ii) solutions to get solutions with pH 4, 5 or 6. Copper adsorption (*q*) was calculated from the difference of the initial concentration (*C*_0_, mg L^−1^) and the final (*C*_f_, mg L^−1^), considering the volume (L)/mass (g) ratio using the eqn (1):

1 *q* = (*C*_0_ − *C*_f_)(*v*/*m*)

Adsorption isotherms were carried out to assess the effect to the initial concentration and temperature on the adsorption process. For it, 200 mg of manganite were added into 125 mL conical flask with 50 mL of solution at different initial concentrations of Cu(ii) (25, 50, 100, 200 and 500 mg L^−1^) at pH 6.0. The temperature of the different mixtures was adjusted at 30, 40 and 50 °C with shaking at 200 rpm during 24 h. The suspensions were filtered with a 0.20 μm film. The samples were extracted from filtrates and determined for Cu(ii) concentration using an atomic absorption spectrophotometer. Langmuir equation was applied to quantify adsorption capacity given in eqn (2):

2 *q*_e_ = *q*_max_ [(*K*_L_*C*_e_)/(1 + *K*_L_*C*_e_)]

In which, *q*_e_ is the equilibrium adsorption capacity of the adsorbent, *C*_e_ is the equilibrium concentration of Cu(ii) (mg L^−1^), *q*_max_ is the maximum adsorption capacity (mg g^−1^) and *K*_L_ is the equilibrium constant of Langmuir (L mol^−1^). The lineal form of eqn (2) can be represented as eqn (3) shows:

3 *C*_e_/*q*_e_ = [(1/*q*_max_)*C*_e_ + (1/*K*_L_*q*_max_)]

Adsorption capacity of manganite for Cu(ii) was evaluated using the lineal form of Langmuir model. All adsorption experiments were performed in triplicate and data were analyzed using one-way ANOVA (*p* < 0.05).

#### 2.3.1. Calculation of thermodynamic parameters

The temperature dependency of adsorption is associated with the total energy change of the system (Δ*H*°) and the Gibbs free energy change (Δ*G*°), these energetic changes are the criteria for the variation of the entropy (Δ*S*°). By these parameters, it can be decided whether adsorption is a spontaneous process or not. In a spontaneous chemical reaction and in others physicochemical transformations, the Gibbs free energy decreases; that is, Δ*G*° becomes negative. A positive Δ*G*° means free energy will increase. This shows the reactions progressing in the opposite direction, that is to say, in the involuntary direction.^16^ The thermodynamics parameters were calculated by the application of the following equations:^17^

4 Δ*G*° = −*RT* ln *K*_L_

5 Δ*G*° = Δ*H*° − *T*Δ*S*°

Based on the equations above, the following van't Hoff equation can be derived:

6 −*RT* ln *K*_L_ = Δ*H*° − *T*Δ*S*°

7 ln *K*_L_ = (Δ*H*°/*RT*) + (Δ*S*°/*R*)

A plot of ln *K*_L_*vs.* 1/*T* is a line. Δ*H*° and Δ*S*° values can be obtained from the slope and intercept, respectively.^16–18^

## 3. Results and discussion

### 3.1. Characterization of manganite

**Fig. 1:**
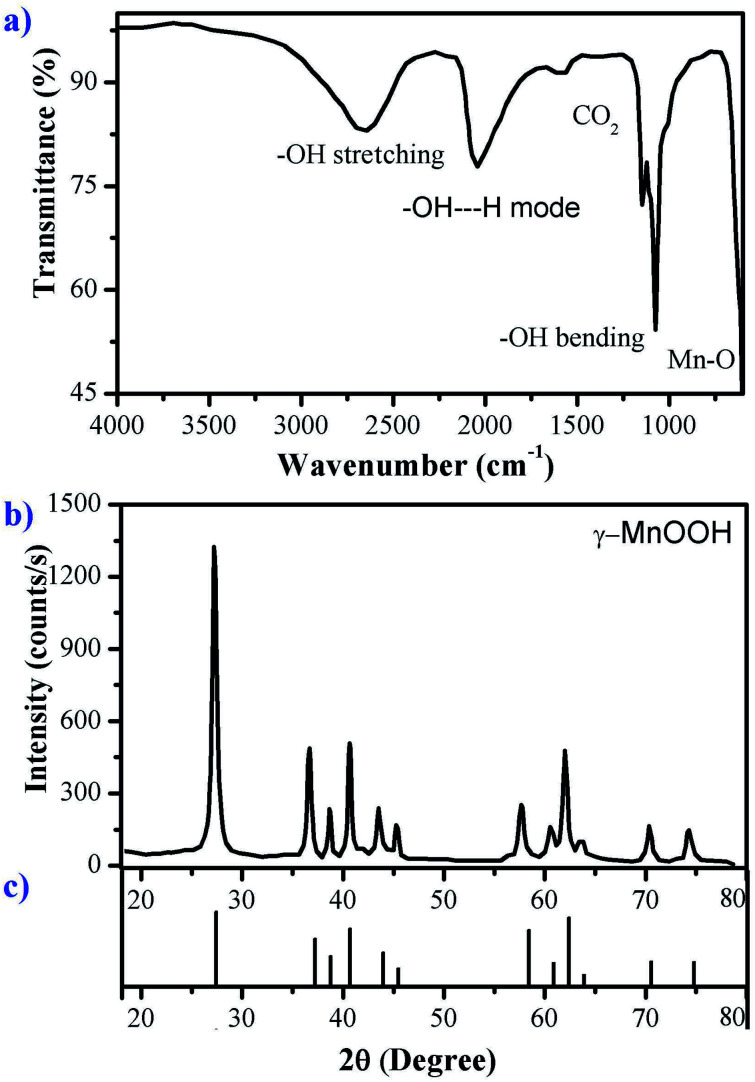
(a) FTIR-ATR spectrum, (b) X-ray diffraction pattern of manganite sample and (c) diffraction pattern of MnOOH (JCPDS 41-1379).

The FTIR-ATR spectrum of manganite is shown in Fig. 1a. The band at 1590 cm^−1^ is attributed to C–O stretching vibration of absorbed carbon dioxide on the sample, because crystalline materials exhibit a high surface/volume ratio.^19^ The –OH bending modes are observed around 1000–1150 cm^−1^. The lowest-energy band of this group is assigned to the γ-OH mode, and the remaining two represent δ-OH modes.^20^ The broad band at 2600–2700 cm^−1^ is the fundamental O–H stretching band related to the hydrogen bond with and O–H⋯O length of about 2.60 Å in the structure of manganite.^17,19–21^ Another band at 2050 cm^−1^ is also observed, this band could be considered as a combination band of the OH-stretching mode at around 2670 cm^−1^ and the excited lattice mode below 600 cm^−1^. The absorption peaks below 627 cm^−1^ could be attributed to Mn–O vibrations.^19–21^ It has been reported that the presence of –OH groups linked to transition metals has a soft base character promoting chemical adsorption interactions with cations. This type of adsorptive interaction depends on the pH, at acidic pH (0–3) –OH groups could be protonated losing adsorbing capacity; therefore, an optimal adsorption process can be reached in a pH range of 4–6.^22^

All reflections in the XRD pattern of the synthetized manganite shown in Fig. 1b can be readily indexed to the monoclinic phase of γ-MnOOH according to JCPDS file no. 41-1379, shown in the Fig. 1c. So, the results of the XRD analysis indicate that the phase-pure γ-MnOOH was synthesized through the hydrothermal reaction *via* reduction of KMnO_4_. XRD pattern is in accordance with reported synthesized manganite.^16–18^

**Fig. 2:**
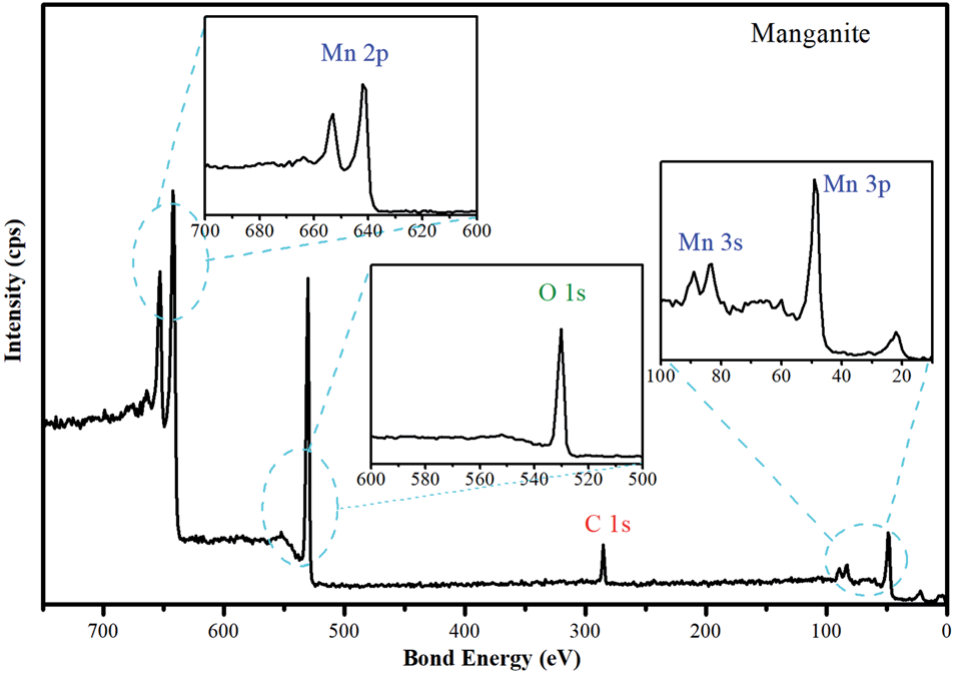
High resolution spectrum of manganite sample. The O 1s, Mn 2p, Mn 3s and Mn 3p spectra of manganite are included.

To investigate the chemical environment in the surface of the manganite sample, one XPS analysis was performed, the results are shown in Fig. 2. In the high resolution XPS spectrum of manganite sample the appearance of peaks of O 1s, Mn 2p, Mn 3s and Mn 3p is observed, which are characteristics of MnOOH.

A crystalline manganite (γ-MnOOH) has been synthesized and characterized using the XPS technique, the surface composition depends significantly on the pH solution. The O/OH group ratio differs between pH 6 and 11, in agreement with the acid/base characteristics of the surface. Below pH 3, on the other hand, drastic changes are observed due to dissolution of manganite. Possibly, the interaction of –OH groups with H^+^ in acidic suspensions is the first step in leading to dissolution.^23^ Accordingly, the crystalline structure of the manganite with adsorbent property is influenced by the pH, therefore, Cu(ii) adsorption studies must be performed in a range of pH 4–6. Alkaline pH will be avoided due to generation of insoluble hydroxides derived from Cu(ii).

**Fig. 3:**
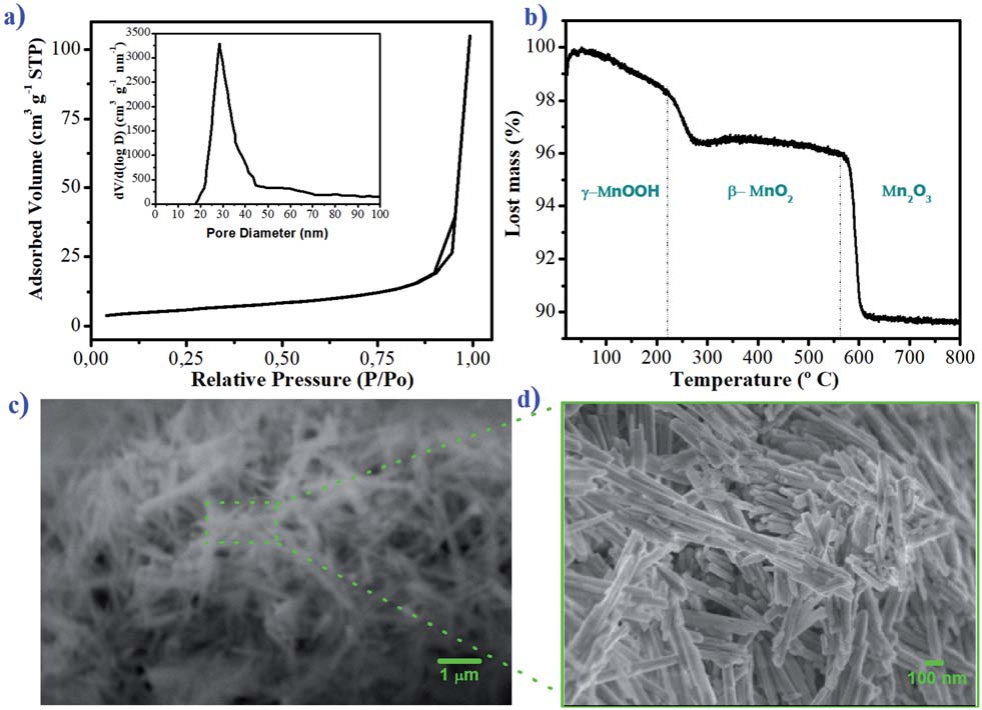
(a) Nitrogen adsorption–desorption isotherms, (b) TG curve, (c) SEM image and (d) FESEM image of manganite sample. Pore size distribution curve is shown as inset of (a).

Adsorption of a sorbate on an adsorbent material is primarily due to interactions of guest molecules with atoms of the adsorbent walls, so it seems logical that a high surface area, either per unit mass or per unit volume, should be desirable for high uptake.^24^ The specific surface area reported for synthetized manganite was among 22 and 75 m^2^ g^−1^.^15,24^ The specific surface area for the synthetized manganite in this work was 20.22 m^2^ g^−1^, which was calculated from adsorption data by BET model (Fig. 3a). The pore size distribution plot reveals that the manganite has an average pore diameter of 32.18 nm and a pore volume of 0.1627 cm^3^ g^−1^, as it is shown in the inset of Fig. 3a, which was calculated from the desorption data by using the Barrett–Joyner–Halenda (BJH) model. Considering that manganite sample has pores with large size, they should come from pores between individual MnOOH nanorods in their aggregation.

The thermal behavior of the manganite was evaluated by TGA, which is shown in Fig. 3b. The thermogram indicates a mass loss of 3.6% at 240 °C, related to the oxidation of manganite (γ-MnOOH) to pyrolusite (β-MnO_2_) and to the loss of adsorbed water. Subsequently, another loss of mass of 10% at 595 °C corresponds to the transformation of β-MnO_2_ to Mn_2_O_3_. This thermal behavior for manganite has been observed in another investigation.^24^

The results of TGA allow to know the different chemical compounds of manganese oxide produced by the manganite when elevating the temperature. Each of these species will have unique adsorption characteristics attributed to its physicochemical structure. The effectiveness of the adsorption of Cu(ii) ions could be compared when using manganite or pyrolusite, however, this is not the objective of the present work.

The morphological and structural characteristics of manganite are evaluated by SEM and FESEM, and the results are presented in Fig. 3c and d. At a microscale level, the manganite particles are organized forming like-whisker aggregates (Fig. 3c). At a nanoscale level (Fig. 3d), the particles appear as uniformly rod-like nanomaterials. The length of the rods ranges from 200 nm to 1000 nm, with a width of 13 nm. This type of nanostructure could have freedom of rotation and orientation in media with agitation, facilitating the adsorption processes on its surface.^25^ The nanometric size of the diameter of the manganite particles as well as their surface area and pore volume are required parameters to promote the entrance, diffusion and adsorption of metal ions. Various systems used for adsorption of metal ions based on zeolites and activated carbon, show similarity in the magnitudes of these parameters.^25,26^ These properties can also be related to the thermodynamic and kinetic dependence of the adsorption of the ion on the surface of the material.

### 3.2. Adsorption studies

#### 3.2.1. Influence of contact time and solution pH

**Fig. 4:**
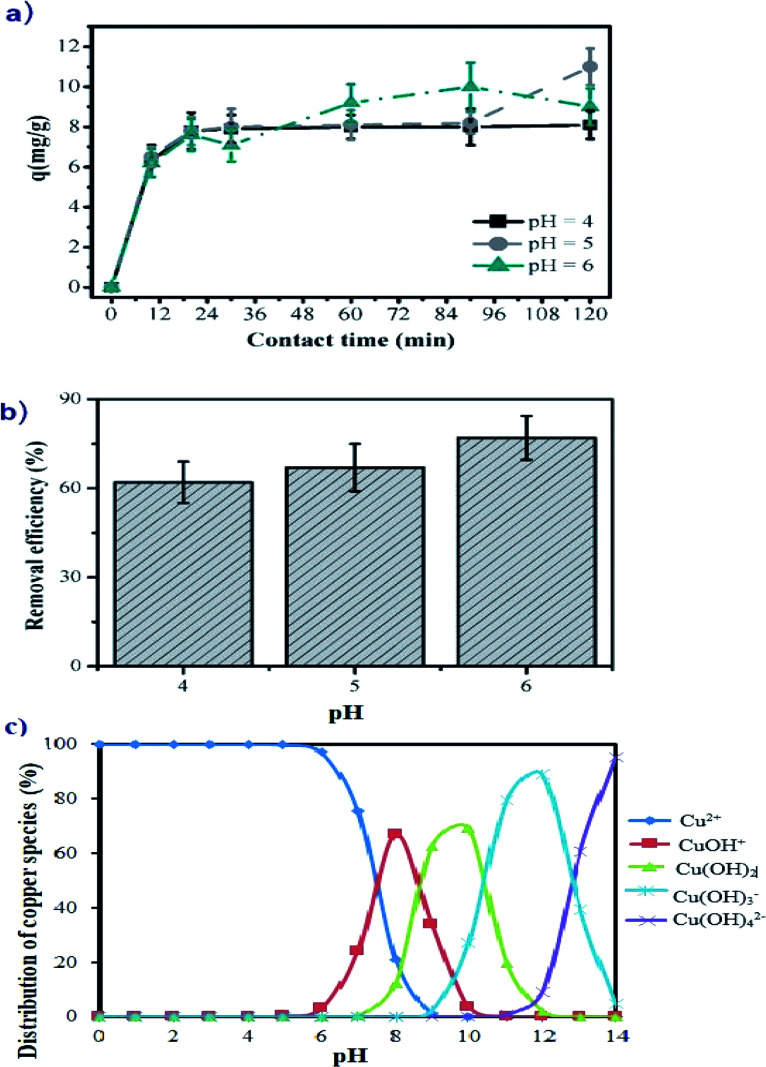
Adsorption of Cu(ii) ions on the manganite surface showing the influence of: (a) the contact time at different pH values; (b) pH of solution at 1 h of contact time and (c) copper species distribution in aqueous solution (modelling with visual MINTEQ 3.1 software, for 25 mg L^−1^ of copper at 1 atm and 25 °C). The initial copper concentration is 25 mg L^−1^.

The experiments varying the contact time were carried out at 30 °C. The dependency of copper adsorption on contact time at three different pH values is shown in Fig. 4a. The kinetic data indicate that the adsorption of Cu(ii) on manganite occurs quickly in the first 20 minutes of contacting time, and Cu(ii) adsorption of 7.5 mg g^−1^ is registered among values of pH 4 to 6. The fast adsorption of copper cations in this pH region, where the manganite surface is positively charged, suggests that the uptake of Cu(ii) from solution to manganite is mainly dominated by chemical adsorption rather than physical adsorption.^27–30^ After 25 min of contact time, the adsorption equilibrium is achieved registering values of *q* lower than 12 mg g^−1^, again showing that the pH does not significantly alter the adsorption of Cu(ii) ions on the surface of the manganite. For practical applications of water remediation contaminated by copper, the use of manganite as an adsorbent is proposed at times less than 25 min to have higher adsorption efficiency; representing a novel and fast remediation alternative.

The aqueous solution pH is an important factor to control the uptake of Cu(ii) on the manganite surface. As presented in Fig. 4b, the copper removal efficiency by using manganite tends to increase from 63.84% to 76.25% with the initial pH varying from 4.0 to 6.0. At pH 4, the adsorption is lower because the surface of manganite would be surrounded by H_3_O^+^ ions, decreasing the interaction of the Cu(ii) ions with adsorbing sites of the surface by increased repulsive forces. As the pH increased, the overall surface on the manganite tends to charge negatively increasing the adsorption. This kind of interaction between Cu(ii) ions and different adsorbents such as dried sunflower leaves, granulated activated carbon, clays and cashew nut shells have been observed.^8,12,31,32^ The kinetic study of Cu(ii) adsorption on manganite nanorods was only performed at pH values lower than 7 (pH = 4, 5, and 6), since Cu(ii) ions react with OH^−^ ions forming a precipitate of Ca(OH)_2_ at pH ≥ 7, as can be seen in the copper speciation diagram (Fig. 4c).

Statistically significant differences are not determined on the adsorption of Cu(ii) in the range of studied pH (*p* < 0.05), therefore, manganite could be used to remove Cu(ii) ions from industrial wastewater at acid pH, without any alteration of its adsorptive capacity.

#### 3.2.2. Influence of initial concentration of Cu(ii) and temperature

**Fig. 5:**
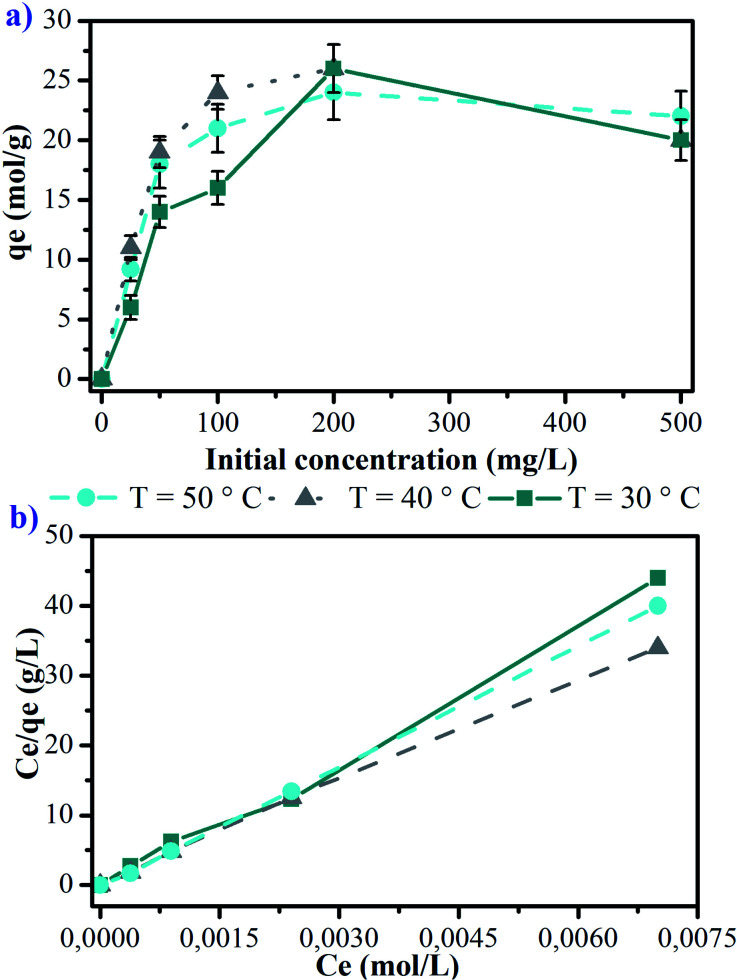
Influence of the initial concentration of Cu(ii) ions and the temperature on the adsorption on manganite: (a) adsorption isotherms at contact time of 24 hours and (b) linear shape of the adsorption isotherms.

The dependence of adsorption capacity for copper on initial Cu(ii) concentrations and experimental temperature are given in Fig. 5a. The adsorption isotherms exhibit similar profiles, which rise sharply at initial concentrations lower than 100 mg L^−1^, then, a plateau phase is obtained at initial concentrations higher than 200 mg L^−1^ of Cu(ii) ions. The study of the effect of temperature indicates that at concentrations lower than 100 mg L^−1^ of Cu(ii) ions values of 16, 21 and 24 mol g^−1^ are observed, for 30, 50 and 40 °C, respectively. Statistically, significant differences among values of *q*_e_ for 50 and 40 °C with respect to 30 °C are found (*p* < 0.05), indicating that a significant improvement in the adsorption of Cu(ii) ions is reached at temperatures higher than 30 °C. On the other hand, it has also been observed that at initial concentrations higher than 200 mg L^−1^, values of approximately 20 mg g^−1^ are reached for all the temperatures of this study, with no significant statistical differences (*p* < 0.05). These results suggest an optimal adsorption of Cu(ii) on the surface of the manganite at temperatures higher than room temperature whereas the initial concentration does not exceed 100 mg L^−1^. The equilibrium of Cu(ii) adsorption reports values of around 20 mol g^−1^ when initial concentrations higher than 200 mg L^−1^ are used, not showing a direct relationship with temperature, indicating that the adsorption process is stable in a defined temperature range, related to chemisorption processes.

In general, the processes of adsorption by physical interactions are modified to increase the temperature, decreasing the amount of adsorbent in the surface.^24,28–30^ In this way, the use of manganite could be a remediation strategy for cases of water contaminated with Cu(ii), keeping its adsorbent capacity at temperatures higher than those environmental ones.

The adsorption capacities of manganite for Cu(ii) were also evaluated using the lineal form of Langmuir equation (eqn (3)), and they are represented in Fig. 5b for 30, 40 and 50 °C. The Langmuir parameters are presented in Table 1. The values of the linear regression coefficients (*R*^2^) for the different evaluated temperatures indicate that copper adsorption experiments on manganite are well fitted by the Langmuir isotherm. The maximum adsorptive capacity is found to be 11.926 mg g^−1^ at 40 °C, however, significant statistical differences were not determined for other temperatures, considering an initial Cu(ii) concentration of 25 mg L^−1^.

**Table 1:** Langmuir parameters for the adsorption of Cu(ii) ions on the manganite surface^a^

Temperature (°C)	*K*_L_ (L mol^−1^)	*q*_max_ (mg g^−1^)	*R* ^2^
30	75.581 ± 28.231*	9.199 ± 1.272	0.996 ± 0.091
40	21.669 ± 1.842	11.926 ± 1.453	0.999 ± 0.095
50	9.183 ± 1.506	9.867 ± 1.862	0.993 ± 0.098

a The average value and the standard deviation are shown for three independent experiments. *p < 0.05.

The values for the equilibrium constant of adsorption process (*K*_L_, pH 6.0) are higher than 1.0, indicating that the equilibrium is displaced towards the adsorption, and not towards the desorption of the Cu(ii) ions on the manganite surface. At 30 °C, the adsorption process registers a *K*_L_ value of 75.581 L mol^−1^ being statically significant compared with the values of *K*_L_ for 40 and 50 °C (*p* < 0.05). Therefore, at 30 °C a higher amount of Cu(ii) ions are adsorbed on the surface of the manganite, however, increasing the temperature, the diffusion of the ions in the manganite–aqueous solution interface also increases, increasing the amount of ions chemisorbed, thus, there is a balance of the adsorbent capacity by modifying the temperature.

The thermodynamic parameters calculated from the adsorption isotherms generate information about the progress of the reaction of adsorption on the material.^33^ The values of these parameters are compiled in Table 2. For any adsorption process the changes in Δ*H*° and Δ*S*° mean, respectively, energy requirements and the randomness of the system components, this results in the transference of the copper ions from solution to the surface of manganite.

**Table 2:** Thermodynamic parameters for the Cu(ii) ions adsorption on the manganite surface at pH 6 varying the temperature of adsorption process

Temperature (°C)	Δ*G*° (kJ mol^−1^)	Δ*H*° (kJ mol^−1^)	Δ*S*° (J mol^−1^ K^−1^)
30	−28.311 ± −4.562	−86.000 ± −12.563	−190.665 ± 32.587
40	−25.993 ± 3.128		
50	−24.516 ± −3.063		

The Δ*G*° values are found to be −28.311, −25.993, and −24.516 kJ mol^−1^ for 30, 40 and 50 °C, respectively. The negative values of Δ*G*° indicate that the adsorption process of copper ions on manganite is spontaneous and thermodynamically favorable. The value of Δ*H*° is also negative (−82 kJ mol^−1^), indicating that the adsorption process of Cu(ii) ions on manganite is exothermic in nature. The negative value of Δ*S*° indicates the stability of adsorption process without structural change in the solid–liquid interface. Due to the magnitude of the calculated thermodynamic parameters, it can be evidenced with the results from the present work, that the adsorption of Cu(ii) ions on the surface of the manganite is a spontaneous process that releases energy and that it is not significantly affected by varying the pH in a range of 4–6, and the reaction temperature in a range of 30–50 °C. These thermodynamic parameters are important in the evaluation of manganite as a potential adsorbent of copper ions. The manganite sample synthesized in this work can be used as an adsorbent material for a fast and efficient remediation of water contaminated with such ion.

In order to compare maximum adsorption capacity of manganite with other adsorbents, Table 3 shows a list of different adsorbents. Natural zeolite reports a *q*_max_ of 1.48 mg g^−1^ of copper, which is lower than *q*_max_ obtained using manganite synthesized in this work.^34^ Manganese oxide coated zeolite (MOCZ) was studied as copper adsorbent, the maximum capacity of copper adsorption was 8.20 mg g^−1^, which is slightly lower than that of manganite obtained in this work.^35^ Natural clinoptilolite-rich zeolite powder has *q*_max_ of 14.93 mg g^−1^, and when it is modified with a bio-inspired adhesive, polydopamine (PDA) has *q*_max_ of 28.58 mg g^−1^. In this case, an increase in Cu(ii) ion adsorption capacity was observed, which is attributed to the chelating ability of the PDA on the zeolite surface.^25^ This approach results innovative because manganite could be supported on polymeric materials in order to increase the adsorptive capacity of Cu(ii). In this sense, the generation of matrices in hydrogel state loaded with manganite with capacity to absorb transition metal ions is indeed a topic of interest for another research work.

**Table 3:** Comparison of maximum adsorption capacity of Cu(ii) by different systems

Adsorbent	pH	BET surface area m^2^ g^−1^	*q*_max_ (mg g^−1^)	Fitted isotherm model	Reference
Modified sawdust xanthate	6	—	28.40	Langmuir	26
Zeolite	—	—	1.48	Freundlich	34
Zeolite	5.5	14.65	14.93	Langmuir	25
PDA24h-zeolite	5.5	4.57	28.58	Langmuir	25
MOCZ	6	28.23	8.20	Langmuir	35
Grape bagasse activated carbon	5	1021	43.47	Langmuir	2
Manganite	5	20.22	11.92	Langmuir	This work

**Fig. 6:**
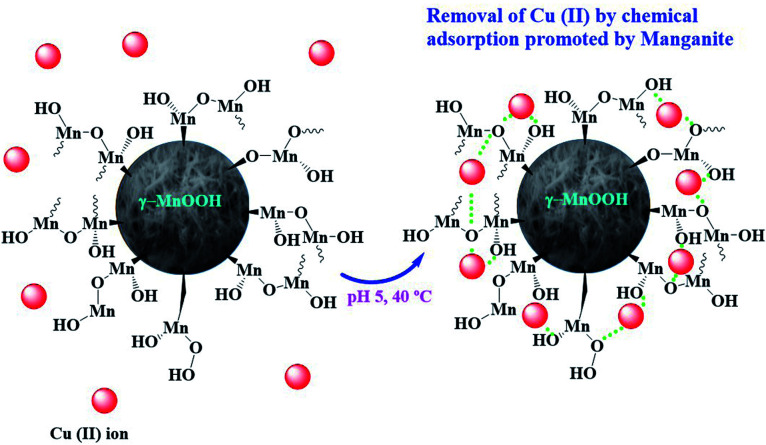
Proposed scheme of Cu(ii) ions chemisorption on the hydroxylated surface of manganite.

Based on the adsorption results obtained, a scheme for Cu(ii) ions chemisorption on the hydroxylated surface of manganite is proposed (Fig. 6).

## 4. Conclusions

The synthesized sample consists of phase pure γ-MnOOH (synthetic manganite), formed by nanorods and it has a specific surface area, pore size and pore volume of 20.22 m^2^ g^−1^, 32.18 nm and 0.1627 cm^3^ g^−1^, respectively. The maximum adsorption capacity of Cu(ii) by manganite was 11.926 mg g^−1^. The adsorption equilibrium results obtained from Langmuir isotherms indicate that the adsorption process is spontaneous with a high preference of Cu(ii) on manganite surface, and that the adsorption reaction is exothermic. The negative value of Δ*S*° suggests the stability of adsorption process without structural change at manganite–aqueous solution interface. With this in mind, manganite could be used as a novel water remediation strategy contaminated with high concentrations of Cu(ii), representing a novel, fast and stable process in the ranges of pH and temperature studied in the present work.

## Conflicts of interest

There are no conflicts of interest to declare.

## Supplementary Material
